# Exploratory Evaluation for Functional Changes of Six-Month Systematic Non-Invasive Electrical Stimulation in a Whole-Body Suit on Children with Cerebral Palsy GMFCS III–V

**DOI:** 10.3390/neurolint17070102

**Published:** 2025-06-30

**Authors:** Tina P. Torabi, Kristian Mortensen, Josephine S. Michelsen, Christian Wong

**Affiliations:** 1 Department of Orthopaedic Surgery, Copenhagen University Hospital, Kettegaards Alle 30, 2650 Hvidovre, Denmark; tina.p.torabi@nord.no (T.P.T.); kristian.mortensen@regionh.dk (K.M.); josephine_michelsen@live.dk (J.S.M.); 2 Department of Orthopedic Surgery, Odense University Hospital, 5000 Odense, Denmark

**Keywords:** child, goals, electrical stimulation, cerebral palsy, prospective study, range of motion, muscle spasticity, posture, neurorehabilitation, movement disorder

## Abstract

Background/Objectives: Spasticity in children with cerebral palsy (CP) can impair motor-related functions. The objective of this exploratory, prospective study was to examine if transcutaneous electrical nerve stimulation (TENS) in a whole-body suit leads to changes in spasticity and other related effects. Methods: Thirty-one children with CP GMFCS III–V, with a median age of 11.0 years (age range of 7–17 years), were consecutively included, and they used the suit with TENS for 24 weeks. The primary outcome was spasticity measured using the Modified Ashworth Scale (MAS). Functional motor-related tasks were evaluated by the Goal Attainment Scale (SMART GAS). The Modified Tardieu Scale (MTS), passive Range of Motion (pROM), GMFM-66, and Posture and Postural Ability Scale (PPAS) assessments were performed. Results: Seventeen subjects (17/31) completed the 24 weeks. Dropout was due to difficulty in donning the suit. The level of overall spasticity, most pronounced in the proximal arms and legs, was reduced according to the MAS, but not the MTS or pROM. Subject-relevant motor-related goals improved significantly in standing/walking and hand/arm function. Changes in the GMFM-66 and PPAS were not significant. Conclusions: Although there were statistically significant but underpowered changes in the MAS after 24 weeks, there were no clinically relevant effects. Exploratorily, we found observer-reliant motor-related functional improvements, which, however, we were unable to detect when trying to quantify them. Donning the suit led to dropout throughout the study. Caregivers need to allocate time, mental capacity and have the physical skill set for donning the suit for long-term use.

## 1. Introduction

Cerebral palsy (CP) is the most common inborn neurological disease in children, with a prevalence of 2.4 per 1000 live births [1]. It is a heterogeneous mosaic of clinical symptoms of non-progressive brain injury stemming from the antenatal, perinatal, or early postnatal period [2]; an apparent symptom of CP is a movement disorder often governed by spasticity. This subsequently affects the musculoskeletal system with, for example, muscular imbalance, joint deformities, and pain [3,4]. This is perceived to lead to decreased motor function and other associated impairments [4]. Several therapeutic spasticity-modifying interventions have been developed to reduce the consequences of these problems, enhance function, and compensate for their disabilities [2,3,5]. These entail exercise therapies, pharmacological anti-spastic agents, and surgical treatments [2,3,5]. Reduction of spasticity can be achieved by localized intramuscular injections of Botulinum toxins as well as general reduction by oral or intrathecal medical agents such as baclofen [6,7]. These therapies can be as disabling as spasticity itself [5,6,7]. The ideal treatment would entail an effective intervention to treat spasticity that is well tolerated with minimal side effects or complications and with a sustained effect, as well as not being invasive [4,8,9]. Treatments today are either short term in effect, time consuming for the child, or with side effects and complications, such as pain from physical exercises, and weakness, fatigue, and drowsiness from medical spasticity reduction. Recent studies suggest that improvement in function, as expressed by the gross motor function and gait, might not be related to spasticity but rather muscle strength [7,8,9]. Others suggest that there is a partial relationship between function and spasticity—even for the more severely affected children, Gross Motor Function Classification System III–V (GMFCS III–V) [10].

**Figure 1:**
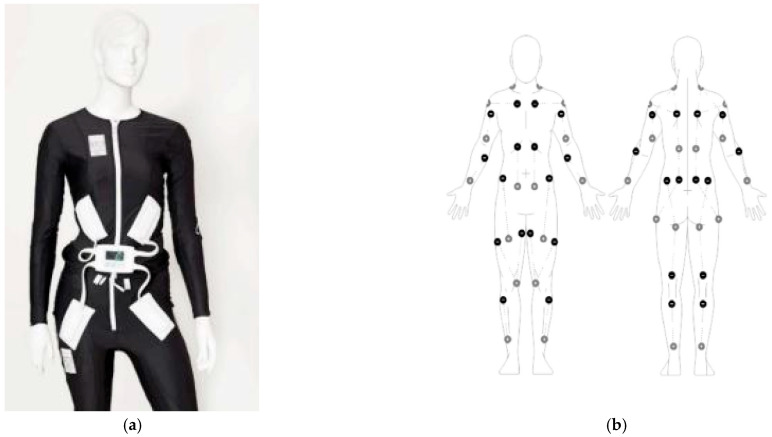
The Mollii suit (**a**) and a schematic drawing of the possible targeted muscles in tailored stimulations according to the pattern of spasticity of the subject using the 58 potential transcutaneous electrical nerve stimulations (**b**).

Transcutaneous electrical nerve stimulation treatment (TENS) for symptoms of spasticity has the tenets of reducing spasticity with only mild potential complications such as skin irritation [11], thus being of interest for CP, since TENS is not invasive, has minimal complications, and can treat symptoms of spasticity and pain. In this study, we examined an assistive device using systematic multi-focal TENS for spasticity reduction incorporated in a whole-body suit. This assistive device was a full-body garment suit with multiple (58) incorporated surface electrodes (whole-body suit), commercialized as the Mollii^®^ suit (Exoneural Network, AB, Stockholm, Sweden) (see Figure 1) [12]. The electrical stimulation was of low intensity and low frequency, which generates a sensory input, but without motor unit recruitment as in functional, electrical stimulation [13,14]. The principle of TENS in the whole-body suit is to stimulate just enough to get the stimulated muscles into a pre-excitatory state. This is provided by stimulation with a frequency lower than 30–35 Hz and with an adequate intensity estimated by clinical evaluation [14,15]. The tenet of the suit is to modify spasticity by systematically stimulating the targeted spastic muscles utilizing reciprocal stimulation, where the antagonist muscles mediate relaxation of the spastic agonist muscles’ reflexes and subsequently lead to decreased muscle stiffness through activation of the disynaptic reciprocal Ia inhibitory pathway. This supposedly enhances the contraction of the agonist muscles and subsequently improves voluntary movements. The tenets of this method are spasticity reduction to enhance function and general stability as well as reduce pain and improve motor function [12,16,17]. Moreover, it is a home therapy-oriented method that is supposed to be applied while performing usual activities of daily living, thus not time consuming as such [13].

In this study, we examined an assistive device using systematic multi-focal TENS for spasticity reduction incorporated in a whole-body suit; thus, the purpose of this prospective exploratory study was to evaluate if the whole-body suit could induce modulation of spasticity and improvements in motor-related functions.

## 2. Materials and Methods

### 2.1. Clinical Rationale for the Study

We set out to examine whether prospectively applied systematic electrical stimulation in a whole-body suit affected spasticity or had other related effects, such as improved motor functions, in a cohort of children with CP GMFCS III–V.

### 2.2. Population

The subjects were pediatric patients with predominantly spastic CP. They were recruited consecutively from three schools for disabled children in the Greater Copenhagen region. The inclusion criterion was spastic cerebral palsy when categorized as GMFCS III–V. Inclusion criteria were evaluated for eligibility according to the charts and clinical evaluation by a pediatric orthopedic surgeon with a special interest in cerebral palsy in children. Exclusion criteria were other disorders affecting the sensorimotor functions as in autism disorder spectrum without spasticity, implanted electrical medical devices, BMI > 35, or other severe concomitant diseases such as cancer, cardiovascular, inflammatory, psychiatric disease, medically dysregulated epilepsy, or hypertension. Subjects were excluded if there was a modification in ongoing pharmacological anti-spastic treatment like botulinum toxin injections or major changes in oral anti-spastic medication, or if there was orthopedic surgery performed three months before and during the test period.

### 2.3. The Whole-Body Suit with TENS

The whole-body suit was worn by the subjects for 24 weeks. The whole-body suit had 58 electrodes that were stimulated at a frequency of 20 Hz, a pulse width ranging from 25–175 µs, and a voltage of 20 V [12]. The settings were adjusted to the child’s age, weight, and level of spasticity of the specific target muscles—as indicated in an overall overview of the stimulated muscles in the Supplemental Materials in Table S1. The whole-body suit (Mollii^®^) and a schematic drawing of the possible targeted muscles are shown in Figure 1. In this study, the settings of the whole-body suit were systematically adjusted to the individual subject by two specialised therapists from the supplier, with the overall aim of providing core stability [12]. Test subjects wore the whole-body suit for one hour every second day in a trial period at home or at their school.

The caregivers, personal therapists, or primary helpers were advised to interact with the child and engage them in their usual physical activity during the one hour of stimulation. Assistance with donning of the whole-body suit was performed by the caregivers at home or by the personal therapist in the school setting to maintain compliance.

### 2.4. Assessments

The subjects were assessed for the level of spasticity over treated/stimulated joints and evaluated using the Modified Ashworth Scale (MAS) and the Modified Tardieu Scale (MTS) [18,19]. The passive Range of Motion (pROM) was evaluated by a goniometer [20]. The Gross Motor Functions Measure-66 (GMFM-66) and the Posture and Postural Ability Scale (PPAS) were performed [21,22]. One single therapist performed all clinical examinations. We applied the Goal Attainment Scale (GAS) using the Specific, Measurable, Achievable, Relevant, and Timed (SMART) principles to evaluate functional changes [23]. The subjects were monitored continuously for adverse effects as well as for any changes in their medications or therapeutic interventions.

#### 2.4.1. Spasticity and Passive Range of Motion

One single therapist performed all examinations before initiation and after weeks 4, 12, and 24 of stimulation in the safe and familiar environment of the school. Testing required supportive assistance from another researcher. This entailed a clinical examination of the MAS, pROM, and MTS of the treated muscles. For the MTS, we evaluated the angle of the catch of the very slow movement (V1) and the very quick stretch (V3) [18]. The effects of the whole-body suit were tested on the following muscles when stimulated. In the proximal lower extremity (LE), we evaluated the muscles of the Iliopsoas, the Quadricep muscle group, the Adductor muscle group, and the Hamstring muscle group. In the distal LE, the muscles of Tibialis anterior and the Gastrosoleus were evaluated. In the upper extremity (UE), we evaluated the Biceps brachii, the Triceps brachii, and the Flexor carpi muscle groups. We measured the pROM using a two-armed goniometer as well as the MAS and MTS in a standardised manner following recommendations [24,25,26].

#### 2.4.2. Goal Attainment Scale Using SMART Principles

Two subject-relevant, specific, unweighted goals were set up at baseline and evaluated by the two primary physiotherapists and/or occupational therapists of the subject before initiation and after 24 weeks. Twenty therapists from the schools were involved. We utilised the GAS using the SMART principles [23,27]. The achieved goals were scaled from the status at baseline and before treatment (−1 or −2), the expected outcome (0), the favourable outcome (+1) and the most favourable outcome (+2). For further analyses, we calculated the t-scores. [28]. The individualised and subject-relevant goals were aimed at improving relevant motor functions of the extremities, exercise tolerance, stability, and tasks of daily living. The SMART GAS evaluations were performed by the primary physiotherapist and/or occupational therapist of the individual subject. These were performed before and after 24 weeks of treatment. Since these goals were subject-specific and thus inherently varied, we categorised the goals systematically following the ‘International Classification of Functioning, Disability, and Health’ (ICF) codes to provide an overview [29]. Table 1 shows an overview of the types of SMART GAS goals.

#### 2.4.3. Gross Motor Functions Measure-66 (GMFM-66) and Posture and Postural Ability Scale (PPAS)

The subjects were evaluated by the GMFM-66 for improvement in function, and the PPAS for stability [21,22]. The GMFM-66 is an assessment tool with 66 questions and five dimensions to evaluate changes in gross motor function. The five dimensions are ‘Lying and Rolling, Sitting, Crawling and Kneeling, Standing, Walking, Running, and Jumping’. The PPAS entails one quantitative assessment and a frontal and sagittal plane qualitative assessment with seven grades to evaluate the ability of four kinds of postural tasks in the supine, prone, sitting, and standing positions. The GMFM-66 was performed before and after 24 weeks of treatment. The PPAS assessments were recorded by video before initiation and after 12 and 24 weeks. The primary physiotherapist and/or occupational therapist of the individual subject performed the GMFM-66 and PPAS examinations. One independent physiotherapist evaluated the video recordings of the PPAS. This particular evaluation was blinded, since we were able to mask the timeline of the video recordings; however, we were unable to blind evaluations for the other clinical tests, since these were performed bedside with the subjects.

### 2.5. Statistical Analysis

The sample size was determined as a convenience sample from the eligible subjects of the three schools. We performed an estimate of sample size using Gpower^©^ (Gpower, Gachenbach, Germany) with a power (1-β) of 0.8, an α level of 0.05, a change in the Modified Ashworth Scale of 0.5 (SD 0.8) in a two-tailed matched paired t-test, and an estimated dropout of 30%; the results indicated that we needed to include 31 subjects for a fully powered study. We also performed a post hoc analysis based on the means and standard deviations of overall spasticity, indicating a power of 0.19 for 17 subjects; see Figure S2 in the Supplemental Materials. Shapiro–Wilk tests, histograms, and QQ plots of the collected data were used to analyse for normal distribution. Data regarding spasticity, the passive Range of Motion, improvement in physical activity, and mobility were analysed with the one-sample Wilcoxon signed-rank test for non-parametric data, and a paired t-test was used for normally distributed parametric data. We considered *p*-values of ≤0.05 statistically significant and applied the appropriate Bonferroni corrections to our analyses. The test of normality, the one-sample Wilcoxon signed-rank test, and the paired *t*-test were performed using the IBM SPSS Statistics, Version 22 (IBM, Richmond, VA, USA).

### 2.6. Declarations

The local committee of ethics approved the study (No H-17004467). All methods and procedures of this study adhered to relevant national guidelines and regulations and the Helsinki Declaration. This included obtaining informed signed consent from all subjects’ legal guardians. Regional registration was obtained following the Danish Data Protection Agency, as stipulated by Danish law J.nr. 2008-41-2240. This study was registered in clinicaltrials.gov before the study initiation (NCT04322825). This work was supported by the European Union’s Eurostars funding program under Grant number E! 10627 Mollii. Data and materials will be made available upon request. There were otherwise no conflicts of interest. The authors contributed adequately to the manuscript for participation. We acknowledge Bandagist Jan Nielsen A/S and Exopulse A/B for aiding with the Mollii suits. Exopulse A/B reviewed this manuscript for scientific accuracy but did not provide input to the content.

## 3. Results

### 3.1. Subjects

Thirty-three patients were invited as subjects to participate as a convenience sample at baseline. Two subjects declined before the initiation of the study. Thirty-one subjects were included before the 24 weeks of intervention, and seventeen completed the intervention. Three subjects had a mixed disorder of spasticity with dystonia (2) and ataxia (1). One subject had metachromatic leukodystrophy with spasticity. The male:female ratio was 1.6:1 at inclusion. The median age was 11.0 years (25 and 75% percentile: 6 and 16 years) with an age range of 7–17 years. A detailed description of the included subjects is shown in Table 2, and the mobility status and treatment history in the Supplemental Table S7.

Fourteen subjects withdrew throughout the study period as follows: six children in weeks 0–4 of the intervention; three children in weeks 4–12; and five children in weeks 12–24. The withdrawals were due to non-compliance (N = 5), garment-related issues (N = 2), epilepsy (N = 1), moving to another region (N = 1), and ungrounded withdrawal or perceived effect not as expected (N = 5). A flow chart of each subject’s history and the timeline of the project is included in the Supplemental Materials (Figure S1).

#### 3.1.1. Spasticity and Passive Range of Motion

We found a significant mean decrease in overall spasticity according to the Modified Ashworth Scale from 2.26 to 1.83 (*p* = 0.001) after the Bonferroni correction (for 11 muscles, *p* = 0.005). The MAS in the muscles of the lower extremity had a significant mean decrease from 2.47 to 1.9 (*p* = 0.001) after the Bonferroni correction (for 8 muscles, *p* = 0.006). The reduction was most pronounced in the proximal LE muscles of the hamstrings and the quadriceps. The MAS of the muscles in the upper extremity had a significant mean decrease from 1.73 to 1.48 (*p* = 0.013) after the Bonferroni correction (for 3 muscles, *p* = 0.016). The changes in spasticity according to the pROM, MAS, and MTS are included in the Supplemental Materials (Tables S2–S5).

The first registered catch/resistance (V3) of the Modified Tardieu Scale during fast joint movement did not change and was not significantly different after 24 weeks when estimated for all treated muscles in general and muscles analyzed separately, except in the hamstring muscles with a change from 92.5° to 100° (*p* = 0.002).

No significant differences were found in the passive Range of Motion (V1). The passive dorsal flexion in the hand changed from 72.5° to 70° (*p* = 0.054); in the quadricep muscles during knee flexion, there was a change from 55° to 80° (*p* = 0.066); and in the hamstrings during the straight leg test, there was a change from 92.5 to 100° (*p* = 0.074).

#### 3.1.2. Goal Attainment Scale Using SMART Principles

**Figure 2:**
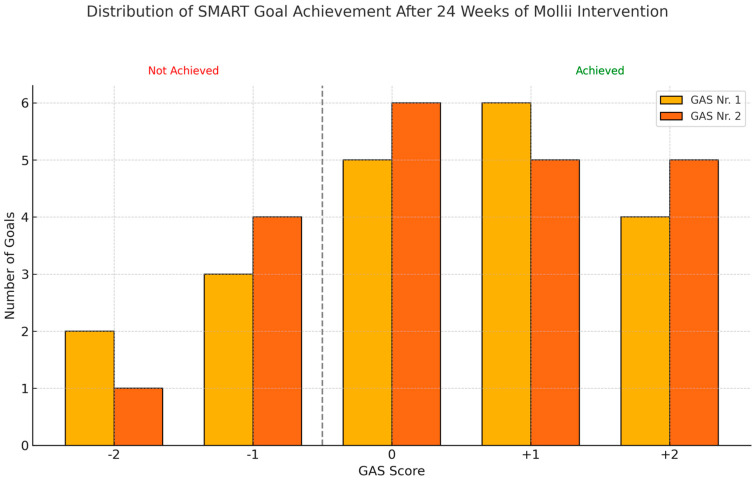
The distribution of achieved SMART GAS goals after 24 weeks of the whole-body suit intervention. The participants had two SMART GAS goals. (GAS Nr. 1 and Nr. 2). GAS scores of 0, +1, and +2 were considered as a goal achieved, and GAS scores of −1 and −2 were considered as goals not achieved.

We found a significant effect in the Goal Attainment Scale. There was an overall significant change in the t-score of 10.3 (SD:8.8) (*p* = 0.001) after the Bonferroni correction (for 12 goals = 0.004). The scores were 38.5 (SD:1.0) and 48.8 (SD:8.6) at baseline and after 24 weeks, respectively. Change in the t-score for goals related to walking improved by 15.1 points; body control by 15.2 points; hand and arm use by 27.4 points; transferring/position changes by 10.2 points; eating/mouth control by 1.6 points; and standing/weight-bearing position by 11.9 points. Figure 2 shows the distribution of achieved goals at 24 weeks according to the GAS.

#### 3.1.3. Gross Motor Functions Measure-66 (GMFM-66) and Posture and Postural Ability Scale (PPAS)

In the GMFM-66, we saw a mean insignificant increase from 27.87 points at baseline to 32.02 (*p* = 0.122). The mean change in Subcategory A: Lying and Rolling changed from 8.17 to 8.33 (*p* = 0.586) and in Subcategory B: Sitting from 13.92 to 14.50 (*p* = 0.443). Subcategories C, D, and E were omitted from the sub-analysis due to data lost at follow-up.

In the PPAS, we registered a slight overall mean increase in the quantitative measures for overall standing up, sitting, and back and front lying down from 13.7 to 14.5 points. For the qualitative evaluation of overall standing up, sitting, and back and front lying down, the measure decreased from 68.3 to 56.5 points. After six months, we found non-significant changes in the quantitative sitting and prone position (*p* = 0.045 and *p* = 0.024), non-significant changes for the qualitative frontal and sagittal prone position (*p* = 0.056 and *p* = 0.078), and no significant changes for the other quantitative and qualitative items using the paired sample *t*-tests (*p* = 0.290–0.890). After three months, no significant changes were seen (quantitative prone position (*p* = 0.080)). We applied the Bonferroni corrections when evaluating for statistical significance (with seven quantitative items, *p* = 0.007 and with 14 qualitative items, *p* = 0.003).

## 4. Discussion

In this exploratory study, we observed a significant reduction in the overall spasticity level according to the MAS. The most pronounced effect was seen in the truncal–near muscles of the thigh and upper arm segments. There were no observed changes in the MTS and pROM. The latter two findings of no effect for the MTS and pROM are collaborated by the study of Bakaniene et al. (2018) [29]. Our finding of a reduction in the MAS is opposed to Ertzgaard et al. (2018) [16]. This might be due to the intervention periods and levels of handicap being shorter and lower for these two studies, respectively, and might explain these differences. Our findings for the MAS might suggest that there would be a relationship between function and spasticity, since we found an improved motor function. However, the high withdrawal rate underpowers our results for the MAS. This might also be influenced by selection bias; thus, our significant reduction in the MAS might be influenced by a type 1 error. However, we did not observe a change in the MTS, thus indicating the presence of this type 1 error. Therefore, our perceived interrelation between function and spasticity should be interpreted as suggestive. When interpreting these changes, we also found that from ‘having a more marked increase in muscle tone through most of the ROM (2)’ changed to ‘a slight increase in muscle tone, manifested by a catch (+1)’. This was statistically significant, but we found the differences to be too small to be clinically meaningful. The differences should also be seen in the context of the limitations in reliability and content validity when using the MAS for measuring spasticity [19,30]. However, we chose the MAS, since it is frequently used and well-known in clinical spasticity evaluation. We compensated for the limitations by using only one specially trained rater for all evaluations to improve the reliability of our measurements. To compensate for the type 1 error of multiple analyses, we utilised the Bonferroni correction in our statistical analyses. A key limitation of this study is the lack of a clearly defined primary outcome, reflecting its exploratory nature. This was due to the complexity of the research question and the variability in available data. In hindsight, a pilot study would have helped refine the design, clarify endpoints, and improve hypothesis framing. This highlights the need for structured outcome definition in early-stage research.

When evaluating for changes in motor function, we observed a significant improvement in the SMART GAS after 24 weeks; thus, there seems to be an effect on function and motor-related goals when using hands and arms, changing positions, and when walking/standing. In previous studies, no functional effects were demonstrated using the SMART GAS, the timed up-and-go test, the action research arm test, and the fast and comfortable gait test [31,32]. Interestingly, both studies had intervention periods of 3 and 6 weeks, respectively, thus indicating that a longer intervention of 24 weeks is needed to achieve an effect on function. Both studies targeted lightly handicapped cerebral palsy patients (GMFCS I–III), which could indicate that the suit has a better effect on more severe cases of cerebral palsy (GMFCS III–V). We acknowledge that when utilising the SMART GAS to describe the functional changes, we are unable to quantify the achieved goals formally. The SMART GAS has commonly been utilised in the pediatric population for similar purposes with good interrater reliability and acceptable content validity [27,33,34]. In this study, the functional improvements were confirmed when interviewing the caregivers, and the goals were set up and evaluated by the subjects’ therapists, who were otherwise not involved in this study, thus supposedly being unbiased. However, their commitment to the subject would potentially affect the evaluation, and we acknowledge that setting up and evaluating the SMART GAS is observer-dependent. In general, the reliance on subjective and observer-dependent measures such as the SMART GAS limits the strength of the evidence, and we acknowledge that it may have influenced the objectivity of outcome assessments, as we involved familiar therapists who, while facilitating patient engagement and consistent follow-up, might have been biased. The absence of more objective, blinded, and standardised quantitative assessments—such as instrumented gait analysis or strength testing—represents a notable limitation of the current study.

We would expect that these observer-dependent improvements in motor function and truncal stability would be reflected in similar improvements in subcategories of the GMFM-66 and PPAS, as these tests also reflect motor function and core stability; however, we found no significant overall motor changes or changes in the evaluated subcategories. Thus, the improvements measured using the SMART GAS might have been too small or not present at all to be acknowledged in the GMFM-66 or PPAS. This was also demonstrated by Bakaniene et al. (2018), who found positive but insignificant changes in the standing, walking, running, and jumping dimensions of the GMFM-88 [29]. We found improvements in the quantitative prone and sitting stability of the PPAS, but these were non-significant after the Bonferroni correction. In a study by Westerlund et al. (2014), balance and trunk stability improved [33], but we were unable to detect significant changes in the PPAS and GMFM-66 after 24 weeks of intervention. In this study, we aspired to be as close to the ‘real-life situation’ as possible by using this home-based therapy. We experienced that having an intervention period of 24 weeks reveals the challenges of home-based TENS therapy, which relies on caregivers’ participation and commitment. This is reflected in treatment compliance and the withdrawal rate of the study. Fourteen out of 31 included participants withdrew from the study. There were multiple reasons for the high number of dropouts. The expected time of usage was 1 h every second day, which amounted to an expected usage of 720 min per calendar month. We accepted that there might be periods of discontinued use of the suit due to, for example, other ‘accidental’ diseases such as pneumonia. While detailed usage data (e.g., hours worn, missed sessions) were not collected, participant compliance was assessed through interviews, and five subjects were excluded due to poor adherence, as noted in the Supplemental Materials section. Two participants withdrew due to garment-related issues, where the parents were unsure if the suit functioned adequately due to technical problems. Three others were excluded as the expectations of the effect of their suits were not met. We interviewed the parents (and therapists), who performed the task of donning the suits; our overall impression was that donning the suit, typically in the morning, was difficult, especially for busy families with, for example, other siblings to attend to. The families expressed that they had had difficulty mustering additional resources for using the suit, even though they had seen an effect for their child. This was independent of factors such as socioeconomic status. Difficulty in donning was the main reason for the low compliance and subsequent dropout from the study. We offered participants the options of both the self-administered donning of the suit and the donning by the participants’ personal therapists to optimise compliance. The personal therapists developed a method of donning from 20 min by two therapists down to 10 min using a special fabric sheet to wrap arms and legs into the relatively tight suit. One dropout was due to the perception that it provoked three epileptic seizures; the parents described that the epileptic attacks were directly related to the use of the suit. Two withdrew without any reason but indicated that they were afraid of epileptic seizures as experienced by the previously described participant. One participant moved outside our region; thus, we were unable to monitor that participant’s study parameters. To address the potential influence of comorbid conditions such as epilepsy, we conducted a review of the participants’ changes in epilepsy status. We consulted an independent clinical specialist in epilepsy who was not involved in the study. The review concluded that the use of TENS did not appear to influence the status or severity of epilepsy in affected participants. Thus, we have included these individuals in our main analysis. We have provided additional data in the Supplemental Materials (Supplemental Analysis: Epilepsy Status and TENS Exposure) outlining any changes in epilepsy status observed during the intervention period. Economic income and educational level did not influence compliance when examined statistically. Our high withdrawal rate was not seen in the study of Bakaniene et al. (2018), with three weeks of intervention, but was closer to Ertzgaard et al. (2018), with compliance in 17 out of 27 subjects after six weeks of intervention [16,29]. This signifies that donning is a major obstacle for this home-based therapy. As mentioned, we offered both self-administered donning of the suit or donning by the subjects’ therapists in order to ‘make life easier’ for the caregivers and to maintain compliance.

## 5. Clinical Implications/Limitations/Future Directions

For future studies, we suggest improvements in study design; this would entail either a parallel control group with the suit only and no stimulation or a cross-over design as in Ertzegaard et al. (2018) [16], a sham suit, or a usual care-only group. Our study design restricts our ability to attribute observed improvements solely to the intervention. Our study signifies itself by a longer follow-up that revealed no sustained benefits and highlighted a high dropout rate. For future studies, we recommend improvements in study design. These could include—as mentioned above—either a parallel control group using the suit without stimulation or a suit with a crossover design. Also, to improve spasticity evaluation, we propose to quantify these measurements instead of using clinical evaluation, thus providing higher reliability. We tried to quantify spasticity using the Neuroflexor© (Aggero MedTech AB, Älta, Sweden) for spasticity evaluation [34], but this was not feasible for children with CP GMFCS III–V, and we had to abandon this and rely on clinical evaluation instead. Likewise, we were unable to evaluate muscle strength, since our subjects were unable to cooperate with this either. The interrelationship between strength and function would have been interesting, since it is suggested to play a major role in function [6]. We also acknowledge that allowing children to continue with physiotherapy and other interventions during the study introduces the potential for co-intervention bias, particularly as these factors were not controlled for or included in the statistical analysis. However, all participating children attended special schools that provided a controlled environment with consistent routines and structured support. This setting may have helped reduce variability in exposure to external interventions. Additionally, therapists were encouraged to report any significant changes in therapy or intervention to the research team, allowing for ongoing monitoring throughout the study period.

We have earlier sought this as an ideal treatment as effective, well-tolerated and without side effects. The prerequisites for using this whole-body suit were that caregivers could allocate time and that they had the stamina and the physical skillset for donning the suit. However, the whole-body suit in this study was, alas, not well tolerated in the sense that donning was difficult; thus, subjects’ and caregivers’ compliance deteriorated during the 24 weeks, resulting in high dropout from the study. This also significantly limits the statistical power of our findings and highlights important concerns regarding both the feasibility and acceptability of the intervention.

In conclusion, for this exploratory study, we were unable to detect a powered effect on spasticity and quantitative improvement in motor function of the whole-body suit for children with CP GMFCS III–V. Our results are underpowered due to the level of drop-out from the study, which we ascribe to difficulty in donning the suit. Although we found observer-reliant functional improvements, we were unable to detect these improvements when trying to quantify them.

## Figures and Tables

**Table 1:** Overview of the SMART GAS goals. Types of goals according to ICF codes, evaluated by the Goal Attainment Scale, and grouped by ICF components.

Component	ICF Code	Description	Frequency
**Body Functions**	b455	Exercise tolerance function	2
	b735	Muscle tone function	3
	b770	Gait pattern function	1
**Activities**	d410	Changing basic body position	4
	d4104	Maintain body control	2
	d415	Weight-bearing	3
	d440	Fine hand use	1
	d445	Hand and arm use	6
	d450	Walking	4
	d455	Moving around	2
	d465	Moving around using equipment	2
**Body Structures**	s510	Improving drooling	1

**Table 2:** Subject characteristics. Age (in years). Sex (M = male; F = female). Wgt. (weight in kg). Hgt. (height in cm). GFMCS (Gross Motor Function Classification System). Classification (topographic). * Data lost in follow-up.** The participant had predominant spasticity.

Patient	Age	Sex	Wgt.	Hgt.	GFMCS	Classification
1	16	M	50	165	III	spastic tetraplegic
2	14	M	30	*	V	mixed ataxic/spastic tetraplegic
3	17	M	50	168	V	mixed dystonic/spastic tetraplegic
4	16	F	47	158	III	spastic tetraplegic
5	9	M	25	129	V	metachromatic leukodystrophy **
6	7	F	18.5	113	V	spastic tetraplegic
7	11	M	23.5	128	V	spastic tetraplegic
8	10	F	30	133	V	spastic tetraplegic
9	8	M	19.5	126	V	spastic tetraplegic
10	16	F	38	150	V	mixed dystonic/spastic tetraplegic
11	12	F	36.5	142	III	mixed dystonic/spastic tetraplegic
12	17	M	48.5	171	V	spastic tetraplegic
13	10	F	20	118	V	mixed dystonic/spastic tetraplegic
14	9	M	24	112	III	spastic hemiplegic
15	17	M	31	129	V	mixed dystonic/spastic tetraplegic
16	9	M	19	118	IV	spastic tetraplegic
17	10	F	24	110	V	spastic tetraplegic

## Data Availability

For further interest, inquiries for data can be sent to the corresponding author.

## Supplementary Materials

The following supporting information can be downloaded at: https://www.mdpi.com/article/10.3390/neurolint17070102/s1, Figure S1: the reasons and timing of participant withdrawals; Figure S2. Posthoc power analysis based on the means and standard deviations of overall spasticity; Table S1: The total number of stimulated muscles for the 17 participants; Table S2: Modified Ashworth. Changes at group Level; Table S3: Modified Tardieu. Changes at group level; Table S4: pROM. Changes at group level; Table S5: Passive range of motion; Table S6: Supplemental Analysis: Epilepsy Status and TENS Exposure; Table S7: Mobility status and treatment history.

## Abbreviations

The following abbreviations are used in this manuscript:

TENS	Transcutaneous electrical nerve stimulation
Whole-body suit	Full-body garment suit with incorporated surface electrodes
CP	Cerebral palsy
GMFCS III–V	Gross Motor Function Classification System III–V
SD	Standard deviation
V1	Angle of the catch of the very slow movement of the Modified Tardieu Scale
V3	Angle of the catch of the quick stretch of the Modified Tardieu Scale
pROM	Passive Range of Motion
MAS	Modified Ashworth Scale
MTS	Modified Tardieu Scale
LE	Lower extremity
UE	Upper extremity
GAS	Goal Attainment Scale
SMART	Specific, Measurable, Achievable, Relevant, and Timed (SMART) principles
GMFM-66	Gross Motor Functions Measure-66
PPAS	Posture and Postural Ability Scale
