# Supplementary material for: Exploratory Evaluation for Functional Changes of Six-Month Systematic Non-Invasive Electrical Stimulation in a Whole-Body Suit on Children with Cerebral Palsy GMFCS III–V
Source: Neurol Int. 2025 Jun 30;17(7):102. doi: 10.3390/neurolint17070102 (PMC12298194; doi:10.3390/neurolint17070102)
Supplement: Supplementary file 1 [file neurolint-17-00102-s001.zip › neurolint-3654023-supplementary.pdf]

Supplemental material:

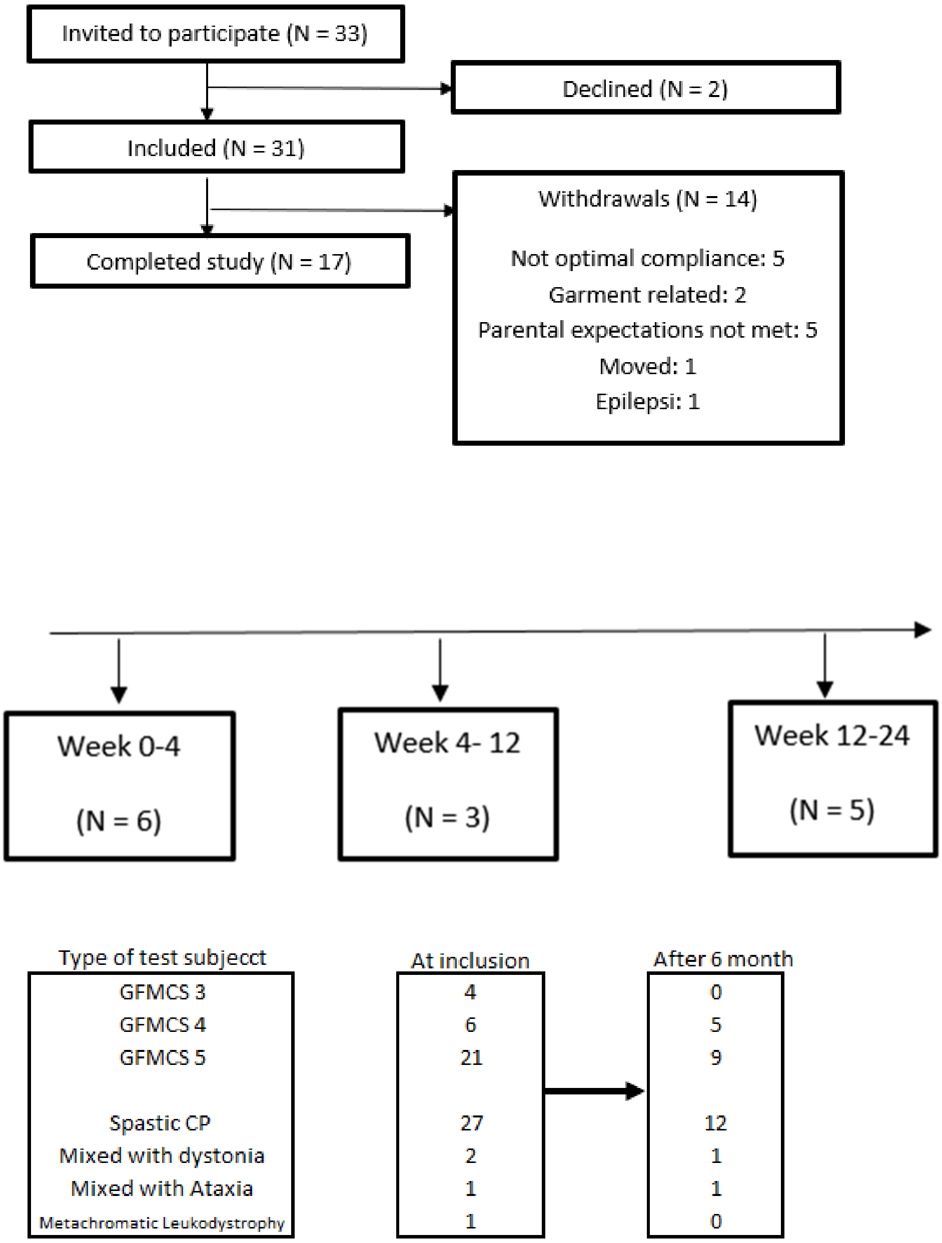

Figure S1. Description of withdrawals during the study regarding reasons of withdrawal (2A↑), timeline (2B→) and type of test subjects (2C↓).

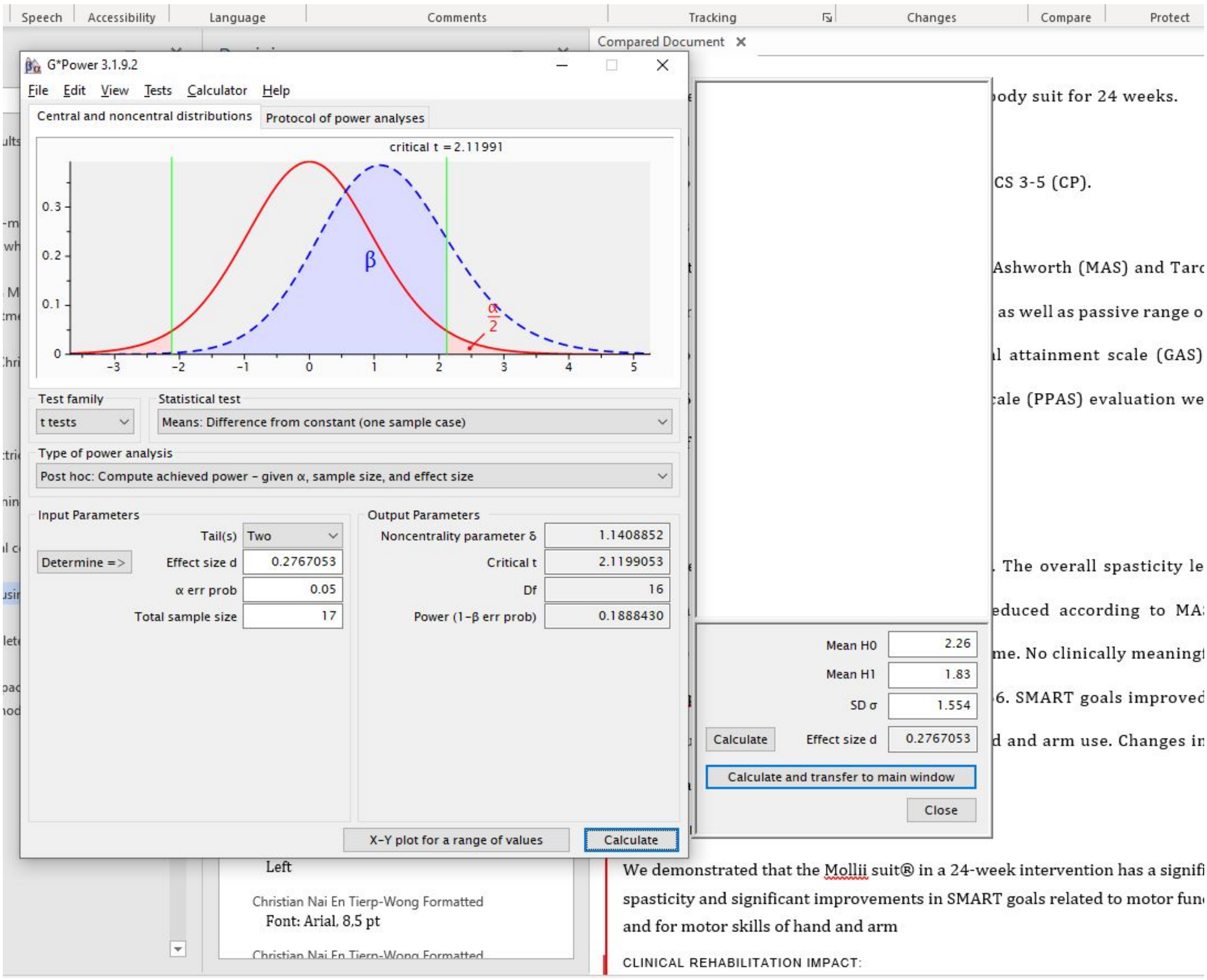

Figure S2. Posthoc power analysis based on the means and standard deviations of overall spasticity.

Table S1. The total number of stimulated muscles for the 17 participants.

Stimulated muscle	Ratio of patients stimulated in specific muscle
R. Carpi flexor group	3/17
L. Carpi flexor group	2/17
R. Carpi extensor group	14/17
L. Carpi extensor group	13/17
R. Elbow flexors	5/17
L. Elbow flexors	5/17
R. Elbow extensor	10/17
L. Elbow extensor	9/17
R. Pectoralis-frontal deltoideus	9/17
L. Pectoralis-frontal deltoideus	9/17
R. Pectoralis-Trapezius (upper)	2/17
L. Pectoralis-Trapezius (upper)	2/17
R. Trapezius upper-lower	6/17
L. Trapezius upper-lower	6/17
R. Deltoideus group	8/17
L. Deltoideus group	7/17
R. Triceps-Trapezius (upper)	3/17
L. Triceps-Trapezius (upper)	3/17
R. Rectus abdominis	17/17
L. Rectus abdominis	17/17
R. Latissimus dorsi (upper)	13/17
L. Latissimus dorsi (upper)	13/17
R. Latissimus dorsi (lower)	2/17
L. Latissimus dorsi (lower)	3/17
R. Hipflexor group	6/17
L. Hipflexor group	6/17
R. Gluteus maximus	7/17
L. Gluteus maximus	10/17
R. Gluteus group (med, max)	7/17
L. Gluteus group (med, max)	4/17
R. Quadriceps	11/17
L. Quadriceps	13/17
R. Adductor group	3/17
L. Adductor group	1/17
R. Hamstring group	6/17
L. Hamstring group	4/17
R. Tibialis Ant.	8/17
L. Tibialis Ant.	9/17
R. Gastrocnemius	9/17
L. Gastrocnemius	7/17

Table S2. Modified Ashworth. Changes at group Level

Time	1 M	%	3 M	%	6 M	%	1 Y
**Hip Flexors**							
Number of joints	28/32		24/26		12/16		6/6
Decreased or unchanged Spasticity	24	85.7	17	70.8	12	100	5
Increased Spasticity	4	14.3	7	29.2	0	0	1
**Hip adductors**							
Number of joints	30/32		26/26		16/16		6/6
Decreased or unchanged Spasticity	29	96.7	22	84.6	13	81.2	4
Increased Spasticity	1	3.3	4	15.4	3	18.8	2
**Knee flexors**							
Number of joints	30/32		26/26		16/16		6/6
Decreased or unchanged Spasticity	28	93.3	22	84.6	16	100	5
Increased Spasticity	2	6.7	4	15.4	0	0	1
**Plantar flexors**							
Number of joints	28/32		24/36		16/16		6/6
Decreased or unchanged Spasticity	21	75	15	62.5	12	75.0	4
Increased Spasticity	7	25	9	37.5	4	25.0	2
**Knee ext**							
Number of joints	16/32		16/26		8/16		6/6
Decreased or unchanged Spasticity	15	93.8	14	87.5	8	100	5
Increased Spasticity	1	6.2	2	12.5	0	0	1
**Summary**							
Number of joints	132/160		116/130		68/80		30/30
Decreased or unchanged	119	90.2	90	77.6	61	89.7	24
Increased spasticity	13	9.8	26	22.4	7		

Table S3. Modified Tardieu. Changes at group level.

Time	1 M	%	3 M	%	6 M	%	1 Y	
**Hip flexors**								
Number of joints	12/32		13/26		6/16		2/6	
Improved or unchanged	9	75	9	69.2	2	33.3	2	
Increased Spasticity	3	25	4	30.8	4	66.7	0	
**Hip adductors**								
Number of joints	22/32		19/26		14/16		2/6	
Improved or unchanged	18	81.8	13	68.4	9	64.3	1	
Increased Spasticity	4	18.2	6	31.6	5	35.7	1	
**Knee flexors**								
Number of joints	27/32		24/26		14/16		4/6	
Improved or unchanged	18	66.7	10	41.7	10	71.4	2	
Increased Spasticity	9	33.3	14	58.3	4	28.6	2	
**Plantar flexors**								
Number of joints	28/32		24/26		16/16		6/6	
Improved or unchanged	18	64.3	15	62.5	8	50.0	4	
Increased Spasticity	10	35.7	9	37.5	8	50.0	2	
**Knee extensors**								
Number of joints	4/32		2/26		0/16		0/6	
Improved or unchanged	2	50.0	2	100	0	0	0	
Increased Spasticity	2	50.0	0	0	0	0	0	
**Summary**		%		%				%
Number of joints	93/160		82/130		50/80		14/30	
Improved or unchanged	65	69.9	49	59.8	29	58.0	9	
Increased spasticity	28	30.1	33	40.2	21	42.0	5	

Table S4. pROM. Changes at group level							
**Time**	**1 M**	**%**	**3 M**	**%**	**6 M**	**%**	**1 Y**
**Hip extension**							
Number of joints	26/32		20/26		12/16		6/6
Improved or unchanged	22	84.6	13	65	8	66.7	5
Impaired movement	4	15.4	7	35	4	33,3	1
**Hip Abduction**							
Number of joints	30/32		26/26		16/16		6/6
Improved or unchanged	22	73.4	18	69,2	10	62.5	2
Impaired movement	8	26.7	8	30.8	6	37.5	4
**Hip rotation out**							
Number of joints	22/32		20/26		12/16		4/6
Improved or unchanged	20	90.9	18	90	11	91.7	4
Impaired movement	2	9.1	2	10	1	8.3	0
**Knee extension**							
Number of joints	28/32		22/26		14/16		6/6
Improved or unchanged	27	96.4	21	95.5	13	92.9	5
Impaired movement	1	3.6	1	4.5	1	7.1	1
**Knee flexion**							
Number of joints	30/32		24/26		16/16		6/6
Improved or unchanged	23	76.7	15	62.5	15	93.8	4
Impaired movement	7	23.3	9	37.5	1	6.3	2
**Dorsal extension ankle**							
Number of joints	30/32		25/26		16/16		6/6
Improved or unchanged	24	80.0	19	76.0	9	56.3	4
Impaired movement	6	20.0	6	24.0	7	43.8	2
**Summary**							
Number of joints	166/192		137/156		86/96		34/36
Improved or/unchanged	138	83.1	104	75.9	66	76.7	
Impaired movement							

Table S5. Significant changes in modified Ashworth level (upper), passive range of motion (lower) and modified Tardieu scale (middle), One-sample Wilcoxon signed Rank test.* Significant after Bonferroni correction** Significant for $p<0.05$.

Movement	Stimulated Muscles	Number	Median_baseline	Range	24 weeks of intervention (Median)	Range	P- value
Knee Extension	Hamstrings_Dxt	11	4	0-4	3	0-4	**0.015** **
Knee Extension	Hamstrings_Sin	13	4	0-4	3	0-4	**0.014** **
Knee Flexion	Quadriceps_Dxt	6	4	0-4	2.5	0-4	0.059 ***
Knee Flexion	Quadriceps_Sin	5	3.5	0-4	3	2-4	**0.046** **
Dorsal Flexion ankle	M. Gastrocnemius_Sin	12	3.5	0-4	2	0-4	0.147
Dorsal Flexion ankle	M.Gastrocnemius_Dxt	10	3	0-4	2	0-4	0.443
Dorsal Flexion ankle	M. Soleus_Dxt	10	2	0-4	2.5	0-4	0.256
Dorsal Flexion ankle	M. Soleus_Sin	10	3	0-4	1.8	0-3	0.231
Hip Abduction	M. Adductor Longus	11	3	0-4	1	0-4	0.752
Elbow Extension	M. triceps brachii	6	1	0-4	2	0-2	0.655
Dorsal Fx Hånd	M.flexor carpi radialis	16	0.5	0-4	0	0-4	0.168

Movement	Stimulated Muscles	Number	Mean(SD)_baseline	Mean (SD)_24Weeks	P-value
Dorsal Flexion Ankle	M.Gastrocnemius_Dxt	6	19, 3 (6,1)	18,3 (7,9)	0.785
Dorsal Flexion Ankle	M.Gastrocnemius_Sin	11	18,6 (10,7)	17,3 (11,3)	0.776
Dorsal Flexion Ankle	M. Soleus_Dxt	8	16,1 (16,2)	21,3 (13)	0.233
Knee Extension	Hamstrings_Dxt	11	20 (13,4)	36, 8 (18,2)	**0.011** **
Knee Extension	Hamstrings_Sin	12	0 (0)	29,6 (21,1)	**0.005** *
Knee Flexion	Quadriceps_Dxt	5	52, 9 (52,4)	50,8 (35,7)	0.066
Knee Flexion	Quadriceps_Sin	5	70 (49,5)	69 (27,7)	0.138
Elbow Ekstension	M. Biceps Brachii_dxt	8	63, 2 (45,1)	88,3 (11,7)	0.933
Elbow Ekstension	M. Biceps Brachii_sin	8	80,6 (39,2)	90 (14,6)	0.933
Elbow Flexion	M. triceps_brachii_dxt	2	3,8 (7,5)	40 (0)	0.18
Elbow Flexion	M. triceps brachii_sin	2	17,5 (23,6)	57,5 (10,6)	0.18
Dorsal Flexion Hand	M. flexor carpi radialis_dex	9	30,7 (40)	53,21 (58)	0.214
Dorsal Flexion Hand	M. flexor carpi radialis_sin	13	7,9 (40,5)	33 (32,2)	0.248

Movement	Stimulated Muscles	Number	Median_baseline	Range	24 weeks of intervention (Median)	Range	P- value
Dorsal Flexion Ankle	M.Gastrocnemius_Dxt	10	5	(-20 / 20)	7.5	(-15 / 30)	0.547
Dorsal Flexion Ankle	M. Soleus_Dxt	10	30	(-5 / 10)	20	(-15 / 40)	0.438
Knee Extension	Hamstrings_Dxt	13	-65	(-85 /-10)	-55	(-80 /-30)	0.0291 **
Knee Extension	Hamstrings_Sin	12	-55	(-85 /-20)	-60	(-80 /-30)	0.168
Straightleg	Hamstrings_dxt	9	45	(30-75)	37.5	(10 / 55)	0.102
Straightleg	Hamstrings_Sin	11	50	(30-70)	35	(20 / 70)	0.074 ***
Knee Flexion	Quadriceps_Dxt	8	145	(-45 /155)	137.5	(45 /150)	0.066 ***
Knee Flexion	Quadriceps_Sin	6	142.5	(60-150)	130	(60 /150)	0.18
Elbow Ekstension	M. Biceps brachii_dxt	11	-5	(-20 /5)	0	(-20/-5)	0.317
Elbow Ekstension	M. Biceps brachii_sin	10	0	(-35/5)	-5	(-70/10)	0.416
Elbow Flexion	M. triceps brachii_dxt	5	150	(145/155)	150	(-15 /160)	0.461
Dorsal Flexion Hand	M.flexor carpi radialis_dex	16	87.5	(55/110)	90	(10/100)	0.239
Dorsal Flexion Hand	M.flexor carpi radialis_sin	14	72.5	(-75/100)	70	(-80/95)	0.054 ***

Table S6. Supplemental Analysis: Epilepsy Status and TENS Exposure

A subset of participants (N = 7) presented with comorbid epilepsy. A post hoc evaluation by an independent epilepsy specialist indicated no observable exacerbation or alteration in seizure activity or epilepsy management during the intervention period. Detailed observations and any changes in reported epilepsy status are summarized in *Table Supl6.*

	Name	CPR	Number of Registered Cases During Course	Epilepsy before?	Start of the Mollii suit	First Seizure During Test Period
08KPH	-	-	1	Don't know	6/9-2017	28/11-2017
09NC	-	-	2	No	6/9-2017 – 1/11-2017 // 15/11-2017	Seizures occur after starting at the beginning of both periods. No seizures during break.
19Sp	-	-	2 (more severe than usual)	Yes	7/9-2017	-
13LV	-	-	1	Yes	7/12-2017	26/10-2017
22GK	-	-	3 (possibly more)	No	14/9-2017	From January
26AEH	-	-	2+ (Absences)	Yes	5/9-2017	8/2
31LH	-	-	2	Yes	27/9-2017	23/12/2017, second seizure: 17/1/2018

Table S7. Subject characteristics. Age (in years). Sex (M = male; F = female). Wgt. (weight in kg.). Hgt. (height in cm). GFMCS (Gross Motor Function Classification System). Classification (topographic). Stance/Gait (+ or Ass+ = ability to stand with or without aid/ability to walk with or without aid; − = no walking or standing function). Medication (peroral anti-spastic medicine intake). Baclofen (intrathecal baclofen; too sick = evaluated to benefit from intrathecal baclofen but too sick to for the procedure). Epilepsy (medically treated). PEG (gastric tube for feeding). Personal aids (wearable aids such as AFO—lower leg ankle–foot orthosis; DAFO—distal AFO; and spec. Shoes—custom-made shoes). Assistive devices (wheelchair—electric or manual wheelchairs; standing machine—NF walker; walking frame—behind walker). BTXA (botulinum toxin). Surgery (previous surgery).

Patient	Stance/Gait	Medication	Baclofen	Epilepsy	PEG	Personal aids	Assistive devices	Bot. Tox.	Surgery
1	Ass+/Ass+	Lioresal	-	+	-	AFO	walking frame, wheelchair	+	foot and knee surgery
2	Ass+/-	Lioresal	-	-	-	AFO	Standing, frame wheelchair	+	tendon lengthening
3	Ass+/-	Lioresal	-	-	+	AFO	walking machine, standing frame, wheelchair	+	-
4	+/Ass+	-	-	-	-	DAFO	walking frame	+	tendon surgery and bilat bony foot surgery

Patient	Stance/Gait	Medication	Baclofen	Epilepsy	PEG	Personal aids	Assistive devices	Bot. Tox.	Surgery
5	Ass+/-	Lioresal	+	+	+	DAFO	walking machine, standing frame, wheelchair	+	-
6	Ass+/-	-	+	-	-	AFO	walking machine, standing frame, wheelchair	+	Pelvic surgery
7	-/-	Lioresal	-(too sick)	+	+	AFO+spinal brace	wheelchair	+	-(too sick)
8	Ass+/Ass+	Lioresal	-	+	+	AFO+spinal brace	walking machine, standing frame, wheelchair	+	-
9	Ass+/-	Lioresal	-	-	-	AFO+spinal brace	standing frame, wheelchair	+	-

Patient	Stance/Gait	Medication	Baclofen	Epilepsy	PEG	Personal aids	Assistive devices	Bot. Tox.	Surgery
10	Ass+/-	Lioresal	-	+	+	AFO	standing frame, wheel chair	+	Foot, hand and spinal fusion
11	+/Ass+	-	-	+	-	AFO	walking frame	+	-
12	Ass+/-	-	-	-	+	lycra suit, AFO	walking machine, standing frame, wheel chair	+	-
13	Ass+/-	-	-	+	+	AFO+spinal brace	walking machine, standing frame, wheel chair	+	multilevel tendon lengthening
14	+/Ass+	-	-	-	-	AFO+spinal brace	walking cycle, wheel chair	+	foot surgery
15	Ass+/-	Baclofen	- (too sick)	-	-	Spinal brace, spec. shoe	wheel chair	+	- (too sick)

Patient	Stance /Gait	Medication	Baclofen	Epilepsy	PEG	Personal aids	Assistive devices	Bot. Tox.	Surgery
16	Ass+/-	-	-	+	-	AFO	standing frame, wheel chair	+	- (too sick)
17	Ass+/-	Lioresal	-	+	+	Spinal brace, AFO	standing frame, wheel chair	+	foot and knee surgery
